# Living donor liver transplantation for Budd‒Chiari syndrome with right posterior segment graft and patch plasty using the superficial femoral vein: a case report

**DOI:** 10.1186/s40792-021-01224-5

**Published:** 2021-06-04

**Authors:** Norikazu Une, Kazuaki Tokodai, Norifumi Kanai, Yoshikatsu Saitoh, Mineto Ohta, Kengo Sasaki, Koji Miyazawa, Toshiaki Kashiwadate, Atsushi Fujio, Wataru Nakanishi, Shigehito Miyagi, Michiaki Unno, Takashi Kamei

**Affiliations:** Department of Surgery, Tohoku University Graduate School of Medicine, 1-1 Seiryo-machi, Aoba-ku, Sendai, Miyagi Japan

**Keywords:** Budd‒Chiari syndrome, Living donor liver transplantation, Right posterior segment graft, Superficial femoral vein

## Abstract

**Background:**

In living donor liver transplantation (LDLT) for patients with Budd‒Chiari syndrome (BCS), there are several concerns about reconstruction of the inferior vena cava (IVC) and hepatic veins. Herein, we report the case of a patient with BCS who underwent LDLT with right posterior segment graft (RPSG) and patch plasty for reconstruction of the hepatic venous outflow, using the patient’s own superficial femoral vein (SFV).

**Case presentation:**

A 19-year-old man, who was diagnosed with primary BCS, underwent LDLT. His main hepatic veins were totally obstructed, and membranous stenosis was seen in the IVC. The LDLT donor was his mother; however, liver volumetric analysis showed that only her RPSG was appropriate. In the recipient surgery, 16 cm of the left SFV was harvested and was cut longitudinally and opened. The right hepatic vein (RHV) of the RPSG was anastomosed to the sidewall of the SFV graft. After explantation of native diseased liver was completed, the stenotic and thickened wall of the IVC was widely resected, and a large anastomotic orifice was created. Patch cavoplasty was performed with the RHV‒SFV graft patch. After portal reperfusion started, hepatic venous outflow was satisfactory, and there was no venous graft congestion. Both his postoperative course and his long-term course after discharge were uneventful.

**Conclusions:**

In LDLT for BCS patients, ingenuity is required for the reconstruction of venous outflow. The SFV patch can be safely harvested from liver transplant recipients and is suitable for venous reconstruction. In addition, RPSG is an alternative type of liver graft for LDLT if a conventional right- or left-lobe graft cannot be used.

## Background

Budd‒Chiari syndrome (BCS) is a rare disease involving obstruction of the hepatic venous outflow at various levels from the small hepatic vein to the suprahepatic lesion of the inferior vena cava (IVC) [1, 2]. This hepatic venous obstruction increases hepatic sinusoidal pressure, followed by portal hypertension, which causes liver injury and finally liver cirrhosis. When various treatments for patients with BCS have failed, and liver cirrhosis is decompensated, liver transplantation remains the only curative option [2, 3]. In deceased donor liver transplantation (DDLT) for patients with BCS, suprahepatic caval resection and replacement IVC is the standard procedure. However, due to the absence of an IVC in the liver graft in living donor liver transplantation (LDLT), there are technical difficulties in the implantation procedure in terms of the construction of hepatic venous outflow [3]. In adult-to-adult LDLT, right-lobe graft and left-lobe graft transplants are mainly used. However, this sometimes involves difficulties with limited graft selection, particularly in terms of insufficient graft volume for recipients and a small residual liver volume in the donor. Right posterior segment graft (RPSG) transplant has been introduced in mainly Asian countries as an alternative graft procedure to increase the number of donor candidates safely [4]. We herein report the case of a patient with BCS who underwent LDLT with RPSG and patch plasty for reconstruction of the hepatic venous outflow using the patient’s own superficial femoral vein (SFV).

## Case presentation

**Fig. 1:**
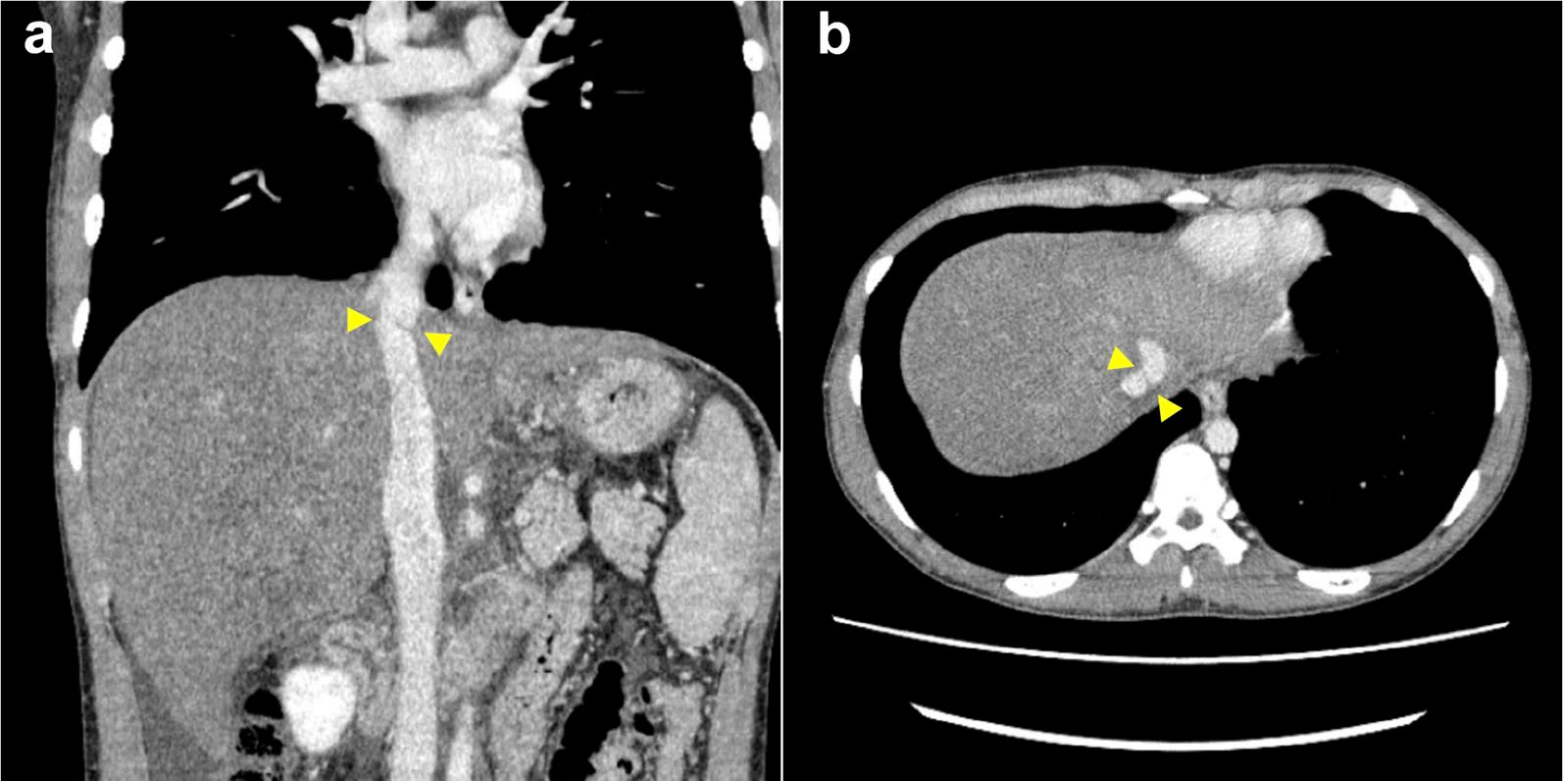
Preoperative abdominal computed tomography (CT) showing the membranous-like stenosis (yellow arrowhead) and non-perfusion of the main hepatic veins (**a** coronal section, **b** axial section)

**Fig. 2:**
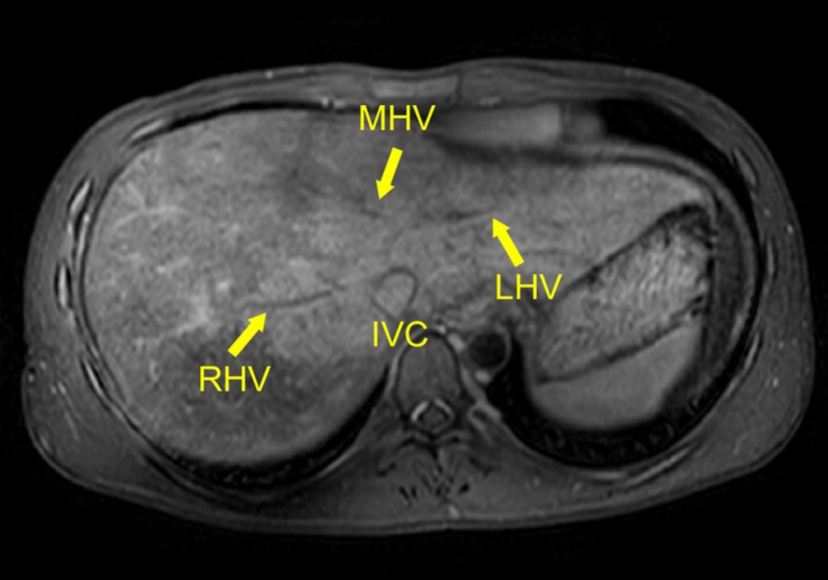
Preoperative abdominal magnetic resonance imaging (MRI) showing obstructed and scarred main hepatic veins. Abbreviations: IVC, inferior vena cava; LHV, left hepatic vein; MHV, middle hepatic vein; RHV, right hepatic vein

A 19-year-old man with no medical history or relevant family history was referred to our hospital because of elevated liver enzyme levels and abdominal distention. An abdominal computed tomography (CT) scan showed that his three main hepatic veins were completely obstructed and that the suprahepatic IVC had stenosis involving a membranous-like structure (Fig. 1a, b). Moreover, the CT scan revealed severe ascites and esophagogastric varices. Magnetic resonance imaging (MRI) showed a lack of patency and scarring in the main hepatic veins (Fig. 2). The patient’s laboratory findings before transplantation showed elevated total bilirubin and decreased platelet and prothrombin time (total bilirubin, platelet, and prothrombin time was 3.8 mg/dl, 150 × 10^3^/ml, 56.2%, respectively). His Child–Pugh score was 11 (grade C), and his Model for End-stage Liver Disease score was 14. He was considered to have no blood disorders that could cause coagulation abnormalities, such as abnormal protein C and S activities. Accordingly, he was diagnosed with primary BCS and decompensated liver failure. Endovascular treatment for the main hepatic veins was considered; however, we recognized that it could be impossible to restore the patency of the hepatic vessels, and we did not perform the preoperative angiography. In addition, his esophagogastric varices were severe. For these reasons, we decided to perform LDLT 5 months after referral.

Donor candidates for LDLT were limited in this case. The eventual donor was his 46-year-old mother, who had no notable medical history and whose blood type was O Rh (+), identical to that of the patient. Based on preoperative CT investigation, we calculated that her estimated whole liver volume was 1047 ml. Her estimated left-lobe graft volume, estimated graft-to-recipient weight ratio (GRWR), and estimated ratio of liver remnant were 303 ml, 0.49, and 71.0%, respectively, which indicated that the size of the left-lobe graft was insufficient for the patient. On the other hand, in the right-lobe graft without the middle hepatic vein, the estimated graft volume was 734.2 ml, and the estimated GRWR was 1.19, which were sufficient for the patient. However, the estimated liver remnant was only 285.7 ml (31%), which was not applicable for our donor selection criteria. Under these mismatches, neither left-lobe graft nor right-lobe graft could be selected. For these reasons, we considered her RPSG, which could provide sufficient liver volume for the patient (estimated graft volume was 609 ml and GRWR was 0.99), and a safe residual liver volume for the donor (estimated ratio of liver remnant was 38.1%). There were no identified abnormalities of the portal vein, hepatic artery, bile duct, or hepatic vein in preoperative CT and MRI.

Donor hepatectomy was performed with a mid-line incision, and liver mobilization was performed using a laparoscopy-assisted technique. During mobilization, the root of the middle hepatic vein and the right hepatic vein (RHV) were identified. Then, anterior and posterior branches of the hepatic artery and portal vein were skeletonized. The transection line was marked on the liver surface according to the RHV and to the demarcation line when the posterior branch of the portal vein was temporarily clamped. Following a hanging maneuver, parenchymal transection was performed using a cavitron ultrasonic surgical aspiration system without occlusion of portal vein inflow. After transection of the liver parenchyma was completely finished, the biliary duct and portal vein of the right posterior branches were cut. Because the right posterior branch of the hepatic artery was thin and it was considered that anastomosis of this artery with recipient hepatic artery increased risks of artery-related complications, the donor’s right anterior branch of the hepatic artery was sacrificed after the artery blood flow in the anterior liver segment was detected by Doppler ultrasound with temporary clamping of the right hepatic artery, and then the right hepatic artery was cut at the root. Finally, the inferior right hepatic vein (IRHV) and RHV were cut, and the liver graft was harvested. The RPSG was flushed with 1000 ml of University of Wisconsin solution from the portal vein. The actual RPSG weight was 570 g.

Recipient surgery was started simultaneously with donor surgery. Prior to the abdominal incision, 16 cm of the SFV was harvested from his left leg for reconstruction of the IVC and hepatic vein. During the operation, there were approximately 3500 ml of ascites, and dense adhesions were seen around the IVC. The native liver was dark and hardened because of the liver congestion. The common bile duct, right and left hepatic arteries, and portal veins were cut, and then all three major hepatic veins, which were thickened and scarred, were cut while preserving the recipient IVC. Explantation of native, diseased liver was completed. The diseased liver weight was 1765 g, and the roots of three major hepatic veins were completely occluded.

**Fig. 3:**
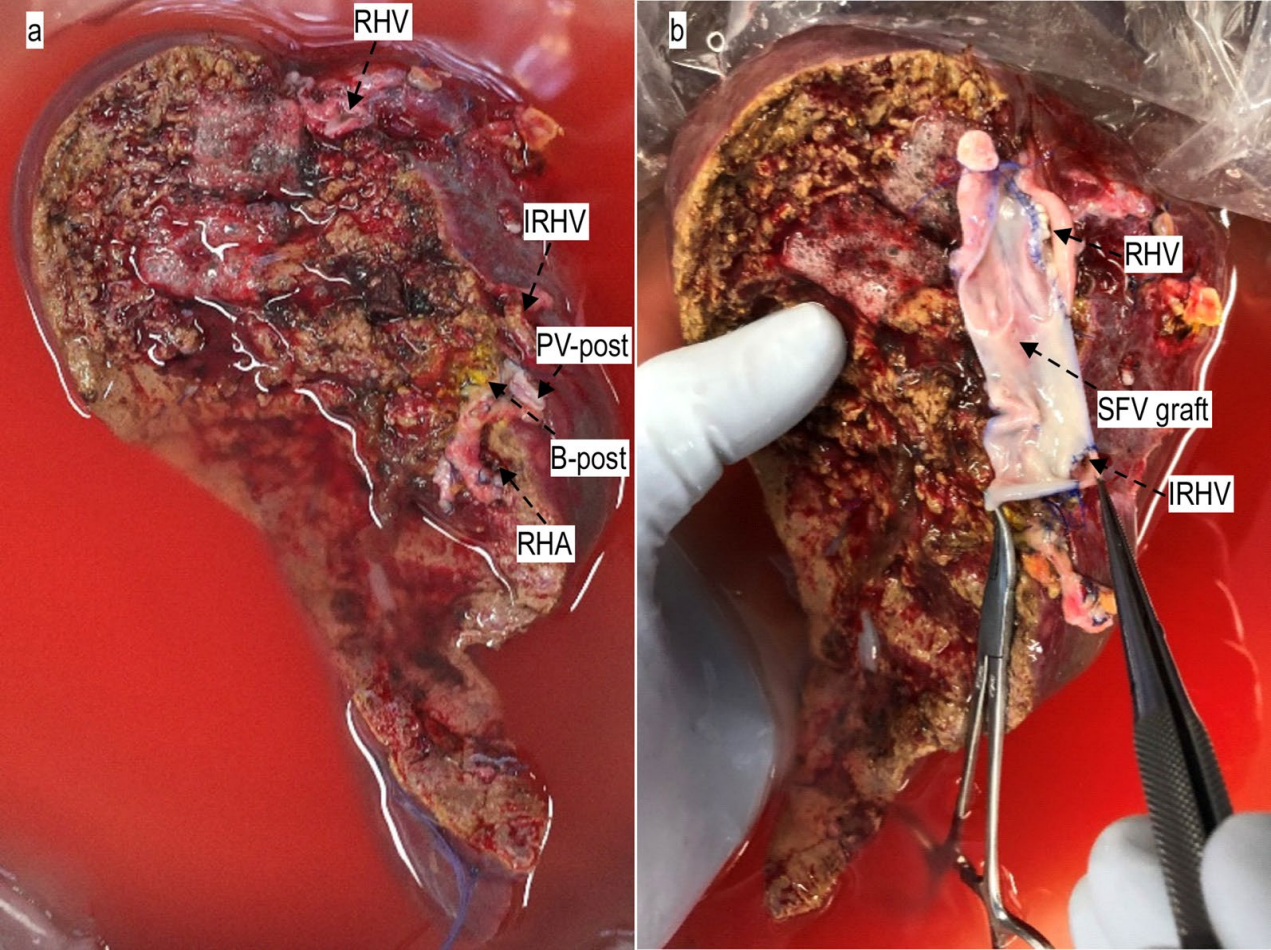
Right posterior segment graft before (**a**) and after (**b**) anastomosis to the SFV graft. Abbreviations: B-post, posterior branch of right hepatic bile duct; IRHV, inferior right hepatic vein; PV-post, posterior branch of right portal vein; RHA, right hepatic artery; RHV, right hepatic vein; SFV, superficial femoral vein

At bench surgery, a longitudinal incision was made from the caudal side of the SFV graft, followed by ligation of the cranial side of the SFV graft. The graft RHV and IRHV was anastomosed to the sidewall of the SFV graft for patch plasty of the IVC (Fig. 3a, b).

**Fig. 4:**
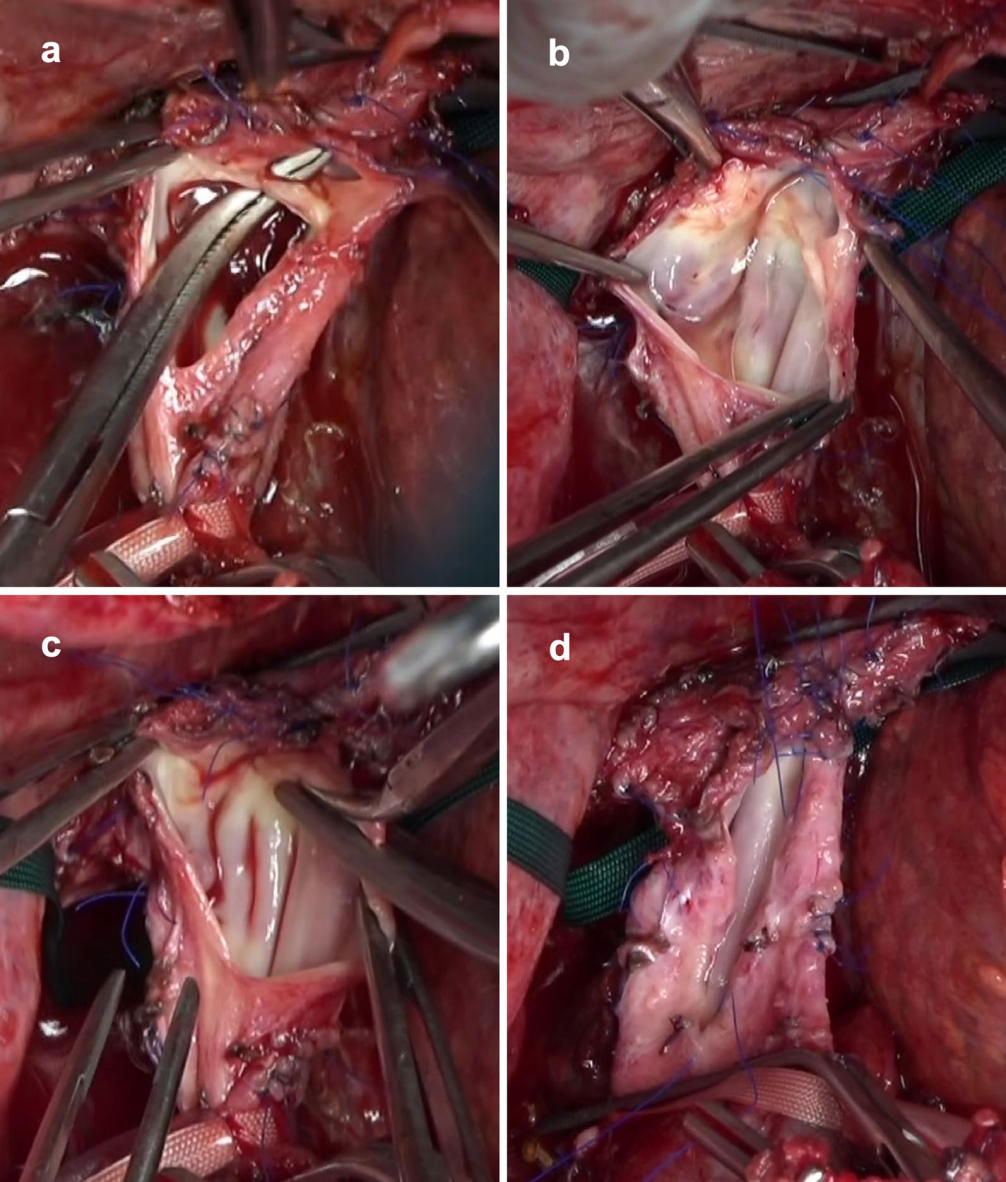
Resection of the membranous web in the suprahepatic inferior vena cava (IVC) and creation of the orifice for patch cavoplasty. **a** Severe stenosis is seen when the suprahepatic IVC was opened. **b** The membranous web in the IVC. **c** Resection of the membranous web. **d** Creation of the anastomotic orifice for patch cavoplasty

For graft implantation, the infrahepatic IVC was mobilized and exposed, and then cross-clamping was performed. Venoveno bypass was not used during graft implantation because the collaterals were well developed, and the hemodynamic parameters were stable after IVC clamping. The thickened anterior suprahepatic IVC was longitudinally cut and opened, and the stenotic lesion of the IVC was identified (Fig. 4a). There were only a few patent millimeters in the IVC due to the membranous web-like obstruction, and there was no IVC thrombosis. The stenotic and thickened wall of the IVC was resected, and then an anastomotic orifice was created (Fig. 4b‒d). As the caliber of IVC orifice did not coincide with the RHV–IRHV–SFV graft, the SFV graft was cut between RHV and IRHV before anastomosis. The RPSG was placed into the recipient, and then patch cavoplasty procedures were performed. The RHV‒SFV graft patch was anastomosed to the IVC orifice using continuous 5-0 prolene sutures. After the graft portal vein was anastomosed to the patient’s main portal vein trunk and portal reperfusion started, Doppler ultrasonography showed satisfactory hepatic venous outflow, without any venous graft congestion. The IRHV with SFV patch was directly anastomosed to the IVC using side-clamping of the IVC after portal reperfusion started. Thereafter, the graft right hepatic artery was anastomosed to the patient’s right hepatic artery. Finally, bile duct reconstruction, involving a choledochojejunostomy, was performed. The surgical time, cold ischemia time, and warm ischemia time were 1028, 235, and 80 min, respectively. The blood loss during surgery was 8486 ml.

**Fig. 5:**
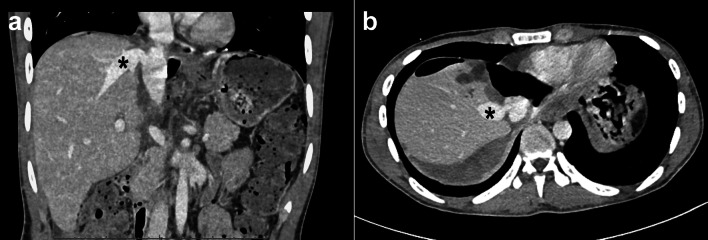
Postoperative enhanced computed tomography showing the right hepatic vein graft (asterisk) and no stenosis in the inferior vena cava (**a** coronal section, **b** axial section)

The donor had no complications and recovered rapidly, and was discharged at postoperative day (POD) 9. The recipient’s postoperative course was also uneventful. Daily Doppler ultrasound revealed patency of the RHV‒SFV graft without venous congestion of the liver, and postoperative CT imaging clearly showed the graft RHV and IRHV with no stenosis in the IVC (Fig. 5a, b). At 13 days after transplantation, the laboratory data showed a slight increase in liver transaminase, and angiography was performed to rule out the hepatic venous outflow block. The postoperative angiography showed no occlusion of the IVC and RHV, and the mean blood pressure of the peripheral RHV, the root of the RHV, IVC, and right atrium were 7 mmHg, 8 mmHg, 7 mmHg, and 4 mmHg, respectively. These findings revealed that the IVC and the graft RHV had sufficient patency, and there was no liver congestion. The increased liver transaminase then normalized spontaneously with no specific treatment. The patient was discharged at POD 28. He has continued to take edoxaban, a direct FXa inhibitor, as a prophylaxis for venous thrombosis. The patient’s condition was good at his last follow-up, 9 months after transplantation.

## Discussion

Budd‒Chiari syndrome is a rare disease and is characterized by an obstruction of the hepatic venous outflow tract, which is located at the level of the small or large hepatic veins, or on the suprahepatic portion of the IVC. This hepatic venous obstruction increases hepatic sinusoidal pressure followed by portal hypertension, which causes liver injury and eventually liver cirrhosis [1, 2]. Differences between Eastern and Western individuals with primary BCS have been reported. Patients with primary BCS in Western countries often tend to have predisposing hematologic conditions, leading to hepatic venous thrombosis, whereas idiopathic cases in Eastern countries are characterized by membranous obstruction of the IVC and hepatic vein [2, 5]. Our case had no particular history or hypercoagulatory condition. Preoperative CT and MR images showed obstruction of the main hepatic veins and membranous stenosis of the suprahepatic IVC. Accordingly, he was diagnosed with primary idiopathic BCS.

For patients with BCS, endovascular treatments, such as angioplasty, stenting, and local thrombolysis, are first conducted. In patients with liver cirrhosis and portal hypertension, a transjugular intrahepatic portosystemic shunt or surgical venous shunt should be considered to decrease the portosystemic gradient [1]. Additionally, anticoagulation therapy is recommended for all patients with BCS to prevent the progression of thrombosis; however, there have been no prospective randomized trials for the efficacy of anticoagulation therapy in BCS [6]. Although there are several treatments for BCS, including anticoagulation and endovascular treatment, liver transplantation is the only curative treatment modality for decompensated patients with end-stage liver disease or acute deterioration. In the present case, we recognized that the patient already had decompensated liver failure, and the hepatic veins might not be sufficiently patent, even if angioplasty or anticoagulation therapy were performed. Therefore, we planned LDLT without angioplasty before liver transplantation.

In DDLT for patients with BCS, suprahepatic caval resection and replacement of the IVC are considered a standard and adequate procedure. In contrast, because the deceased donation rate is low and there is a critical shortage of liver grafts, many patients with BCS in Asian countries undergo LDLT. In LDLT for patients with BCS, the key considerations in surgical procedures are the management of stenosis and occlusion of the IVC and reconstruction of the graft hepatic veins [7]. Due to the mandatory preservation of the donor’s IVC in LDLT, difficult outflow reconstruction is required. There are several reports about reconstruction of the IVC and hepatic veins in LDLT for patients with BCS. If patients with BCS have well-developed venous collateral, the graft hepatic vein is anastomosed directly to the right atrium or supraphrenic IVC without reconstruction of the IVC [8]. On the other hand, if IVC reconstruction is required, cryopreserved autologous vessel grafts, artificial interposition vascular grafts, thrombectomy before hepatic vein anastomosis, or patch plasty for the IVC have been reported in LDLT for patients with BCS [3, 9]. This case had membranous stenosis in the IVC, without thrombosis, and we performed patch plasty for IVC reconstruction using the recipient’s SFV graft. The SFV graft was incised longitudinally to create a large-sized venous graft patch. Although we had to resect the anterior wall of the IVC extensively, reconstruction of the IVC and the hepatic venous outflow tract could be performed completely using the SFV graft, and the RHV‒SFV graft patch has shown good patency over a long period. Harvesting the recipient’s SFV is a safe procedure, and the SFV has an adequate caliber and length [10]. In our institution, when reconstruction of IRHVs and middle hepatic vein (MHV) tributaries in right-lobe graft or RPSG is considered in LDLT, we routinely unify the RHV with IRHVs and MHV tributaries using SFV graft, and unified venous orifice was anastomosed to the IVC [11]. Short- and long-term venous graft patency rates are satisfactory using these techniques.

Additionally, this case underwent LDLT with a RPSG for BCS patient, which has not been reported previously. In adult-to-adult LDLT, right-lobe graft and left-lobe grafts are mainly used. Because of the limited number of donor candidates in many Asian countries, we frequently encounter problems related to an adequate graft size between recipients and donors. Insufficient volume of the left-lobe graft would lead to a small-for-size graft syndrome in recipients. In contrast, the right-lobe graft usually satisfies the liver volume in recipients. However, if donation of the right-lobe graft would induce too small remnant liver volume for the donor, for example, if the remnant liver volume is less than 30%, safe donation is not possible [12]. Although using the RPSG for LDLT is a reasonable and feasible procedure to overcome these problems, RPSG is not widely used in LDLT due to technical difficulties and limited experience [13, 14]. In RPSG procurement, careful donor selection and precise anatomical evaluation are required. In a previous study, Hwang et al. presented that left-lobe volume was less than 30% of whole liver volume, and the presence of type III portal vein (separate posterior branch of portal vein from the main portal vein) will make the possibility of RPSG procurement acceptably high [15]. In addition, Kokudo et al. reported that in patients with arterial anomalies such as the anterior hepatic artery branched off from the posterior hepatic artery itself or very close to the bifurcation of the posterior hepatic artery, it is necessary to consider sacrificing the anterior branch to obtain a single orifice of the right posterior hepatic artery in order to prevent a risk of hepatic artery thrombosis [16]. In a systematic review about using the RPSG, reported by Tagkagi et al., there were no donor deaths after RPSG procurement, and the overall incidences of major and minor complications after RPSG procurement were 5.6% and 34.6%, respectively [17]. Accordingly, it is considered that RPSG for LDLT is acceptable if a conventional right- or left-lobe graft cannot be used. Although several ingenuities and safety procedures were required to perform LDLT with RPSG in this case, the results were satisfactory for both donor and recipient.

## Conclusions

This case report describes a patient with BCS who underwent LDLT with RPSG and patch plasty for reconstruction of the hepatic venous outflow using an SFV graft; this approach has not been reported previously. In LDLT for patients with BCS, marked ingenuity is required to perform optimal treatment under limited conditions. The SFV patch can be safely harvested and is suitable for venous reconstruction.

## Data Availability

Not applicable.

## Abbreviations

BCS: Budd‒Chiari syndrome
CT: Computed tomography
DDLT: Deceased donor liver transplantation
GRWR: Graft-to-recipient weight ratio
IRHV: Inferior right hepatic vein
IVC: Inferior vena cava
LDLT: Living donor liver transplantation
MHV: Middle hepatic vein
MRI: Magnetic resonance imaging
RHV: Right hepatic vein
RPSG: Right posterior segment graft
SFV: Superficial femoral vein
